# Análisis de capacidades institucionales del Ministerio de Salud en Guatemala: restricción democrática, desfinanciamiento, reformas y modelo de atención

**DOI:** 10.1590/0102-311XES027924

**Published:** 2024-11-25

**Authors:** Cristian David Osorio Figueroa

**Affiliations:** 1 Universidade Federal da Bahia, Salvador, Brasil.; 2 Universidade Estadual de Feira de Santana, Feira de Santana, Brasil.

**Keywords:** Gestión en Salud, Creación de Capacidad, Financiación de la Atención de la Salud, Modelos de Atención de Salud, Infraestructura Sanitaria, Health Management, Capacity Building, Healthcare Financing, Healthcare Models, Health Infrastructure, Gestão em Saúde, Fortalecimento Institucional, Financiamento da Assistência à Saúde, Modelos de Assistência à Saúde, Infraestrutura Sanitária

## Abstract

Guatemala es uno de los países de Latinoamericana con mayores inequidades en el acceso a servicios de salud, especialmente en la atención primaria de salud. Múltiples reformas han sido propuestas para solucionar los problemas de accesibilidad sin el éxito esperado, debido a ser experiencias aisladas o la discontinuidad en su implementación. Dada la ausencia de una tradición consolidada en evaluación, no es posible conocer otros factores asociados. De tal forma, el presente trabajo objetivó analizar brechas que inciden en el entorno institucional del Ministerio de Salud Pública y Asistencia Social (MSPAS). Fue utilizado el referencial teórico de análisis de capacidades institucionales para apuntar principales desafíos a ser enfrentados por la institución en su entorno macro-institucional y micro-institucional. Se argumenta que la poca capacidad institucional provocada por los procesos de ajuste estructural debilita la capacidad de respuesta del MSPAS para la garantía del derecho a la salud, situación evidenciada durante la pandemia por COVID-19. El entorno macro-institucional guatemalteco limita el desarrollo de capacidades institucionales al no contar con una tradición democrática consolidada. Sumado, existe una baja capacidad estatal dada la falta de direccionamiento claro sobre sus objetivos, el desfinanciamiento y el enfoque biomédico-hegemónico del modelo de atención que limita el actuar desde un enfoque promocional de la salud. El presente artículo demostró la existencia de limitaciones al desarrollo de capacidades institucionales y la importancia de fortalecer el campo de políticas, planificación y gestión sanitarias.

## Abstract (en)

Guatemala is one of the Latin American countries with the greatest inequalities in access to health services, especially in primary health care. Multiple reforms have been proposed to solve accessibility problems but did not achieve the expected success, either for being isolated experiments or due to their interrupted implementation. Other associated factors are yet unknown, given the absence of a consolidated evaluation over time. Thus, this study aimed to analyze the gaps that affect the institutional environment of the Guatemalan Ministry of Public Health and Social Assistance (MSPAS, acronym in Spanish). The theoretical framework of analysis of Institutional Capabilities was used to point out the main challenges to be faced by the institution in its macro- and micro-institutional environment. It is argued that the low institutional capability caused by the structural adequacy processes weakens the response capacity of the MSPAS to guarantee the right to health, which was evidenced during the COVID-19 pandemic. Guatemala’s macro-institutional environment limits the development of institutional capabilities due to the lack of a consolidated democratic tradition. Moreover, the State holds a poor capacity given the lack of a clear direction regarding its objectives, the lack of funding, and the biomedical-hegemonic approach of the care model that limits action from a health promotion approach. This article demonstrated the existence of limitations to the development of institutional capabilities and the importance of strengthening the field of health policies, planning, and management.

## Resumo

A Guatemala é um dos países latino-americanos com maiores desigualdades no acesso aos serviços de saúde, especialmente na atenção primária à saúde. Múltiplas reformas foram propostas para resolver os problemas de acessibilidade sem o sucesso esperado, por serem experiências isoladas ou devido à descontinuidade em sua implementação. Dada a ausência de uma tradição consolidada em avaliação, não é possível conhecer outros fatores associados. Assim, este trabalho teve como objetivo analisar as brechas que afetam o ambiente institucional do Ministério da Saúde Pública e Assistência Social (MSPAS). O referencial teórico de análise das capacidades institucionais foi utilizado para apontar os principais desafios a serem enfrentados pela instituição em seu ambiente macro e microinstitucional. Argumenta-se que a pouca capacidade institucional provocada pelos processos de adequação estrutural enfraquece a capacidade de resposta do MSPAS para garantir o direito à saúde, situação evidenciada durante a pandemia da COVID-19. O ambiente macroinstitucional da Guatemala limita o desenvolvimento das capacidades institucionais por não ter uma tradição democrática consolidada. Além disso, há uma baixa capacidade estatal dada a falta de um direcionamento claro quanto a seus objetivos, o desfinanciamento e a abordagem biomédico-hegemônica do modelo de atendimento que limita a ação a partir de uma abordagem de promoção da saúde. O presente artigo demonstrou a existência de limitações ao desenvolvimento de capacidades institucionais e a importância do fortalecimento do campo das políticas, planejamento e gestão em saúde.

## Introducción

La pandemia por COVID-19 colocó a prueba la capacidad de respuesta de los Estados a crisis. Fue necesaria la adaptabilidad rápida ante un contexto de incerteza, cuyas respuestas debían ser ajustadas en una conmoción en curso. La heterogeneidad de respuestas dejó grados variados de aprendizajes, éxitos y fracasos ^1^ y dependió de las condiciones socioeconómicas y la capacidad previa del sistema de salud ^2^.

La respuesta fue condicionada por el riesgo de colapso de los servicios hospitalarios debido a la alta transmisibilidad y hospitalizaciones para casos moderados y graves. Fue necesario el refuerzo de medidas de vigilancia sanitaria y epidemiológica. Existieron desafíos para la contratación de talento humano, compra de insumos, equipamiento y, posteriormente, vacunas en contextos de desfinanciamiento de los sistemas sanitarios ^3^.

La crisis causada por la pandemia incluyó la gobernanza y resiliencia de los sistemas públicos para adaptarse, funcionar e innovar para dar continuidad a los servicios públicos y hacerlos extensibles a la población en vulnerabilidad social ^4^. Además, afectó la capacidad relacional del sector público para la formulación de estrategias multisectoriales ^5^.

Al transcurrir el tiempo existió la necesidad de reorganizar el proceso de trabajo de la atención primaria en salud (APS). Fueron incluidas actividades de vigilancia sanitaria, seguimiento a casos crónicos, acciones intersectoriales y el acompañamiento a poblaciones en condiciones de vulnerabilidad social ^6^, en contraposición al modelo de abordaje hospitalocéntrico ^7^ que fue el predominante durante la respuesta inicial.

Estudios recientes colocan que el grado de respuesta de los países dependió de sus trayectorias previas, de la accesibilidad a servicios de salud y de una fuerte institucionalidad democrática ^7^ ^,^ ^8^. Por otro lado, las flaquezas estuvieron asociadas a procesos de debilitamiento de las estructuras de salud pública mediante el desfinanciamiento ^9^, debilitamiento o abandono de la APS y procesos de privatización de los servicios de salud públicos. Quedó en evidencia la falta de cobertura en países europeos o tradiciones no consolidadas de una APS integral y resolutiva ^6^. Estudio previo a la pandemia ya apuntaban una tendencia a ajustes estructurales que debilitaron los sistemas de salud ^10^.

Por lo tanto, una lección de la pandemia es que la capacidad institucional de respuesta dependía de un proceso de acumulación previa y sucesiva que el Estado realiza en su capacidad de gobierno y gestión, la cual no es posible de modificar de forma intempestiva ^11^. Otro elemento es la relación existente con la población y el grado de legitimidad de los actores gubernamentales y los mecanismos de gobernanza ^12^.

A pesar de existir estudios que abordan la capacidad institucional de los sistemas de salud en diversos países ^13^, existen pocos para América Latina ^14^ y específicamente en Guatemala. La situación en Guatemala ha sido abordada desde la discontinuidad de la atención ^15^, los efectos en la salud mental emanados de medidas de distanciamiento social y las condiciones socioeconómicas en determinados grupos de población ^16^. Por ejemplo, la población indígena, las personas trabajadoras del hogar y en situación de calle ^17^, así como la población trans, gay y bisexual ^18^.

Asimismo, fueron apuntados desafíos para la garantía de una atención integral en el contexto de COVID-19. Por ejemplo, la falta de accesibilidad a servicios de atención, baja ejecución presupuestaria, limitada capacidad de testeo, ausencia de programas de investigación y desarrollo, así como, ausencia de mecanismos de participación social ^19^. Todo esto en un proceso de deterioro de las instituciones públicas guatemaltecas acompañado de políticas regresivas en derechos humanos ^20^.

Predominan estudios de políticas específicas de salud siguiendo la tradición científica sanitaria guatemalteca ^21^. Así, este artículo ofrece un análisis actualizado al responder a la pregunta sobre la capacidad institucional del Ministerio de Salud Pública y Asistencia Social (MSPAS) para garantizar el acceso a servicios de salud que condicionó la respuesta a la pandemia. Igualmente, aporta al área de Políticas, Planificación y Gestión, un panorama susceptible de ser profundizado por trabajos empíricos y de análisis política en salud.

En este artículo se argumenta que, aunque exista debilidad institucional y una política regresiva en Guatemala, la posibilidad de una respuesta integral a la pandemia fue perjudicada por la baja capacidad institucional del MSPAS en la gestión, prestación de servicios, financiamiento, organización e infraestructura. De tal forma, se objetiva analizar la entredicha capacidad del ministerio para la garantía del derecho a la salud de la población guatemalteca, mediante el análisis de los componentes del sistema de salud en el contexto de la respuesta a la pandemia por COVID-19.

## Metodología

Estudio de naturaleza mixta. Fuentes documentales y cuantitativas fueron utilizadas para evidenciar el argumento. El alcance del estudio consistió en la prestación de servicios públicos por el MSPAS, subsector público del sistema ^19^.

El referencial teórico utilizado se basó en trabajos previos sobre capacidad estatal. Grindle ^22^, define la capacidad estatal desde un punto de vista instrumental, entendiéndola como la habilidad por parte de las agencias estatales para realizar tareas con efectividad, eficiencia y sustentabilidad.

En contextos democráticos, las capacidades estatales deben estar dirigidas a cumplir los objetivos de políticas resultantes de la deliberación pública. Además, estar dirigidas a la satisfacción de las necesidades de la ciudadanía. Es decir, políticas dirigidas a la generación de valor público, a las cuales deberían adecuarse las capacidades pertinentes ^23^.

Es posible desagregar el análisis en dos niveles: el macro-institucional, cuyo núcleo de análisis es la ausencia total, parcial o presencia de un entramado institucional y sus condiciones externas necesarias para la constitución de los objetivos institucionales. El micro-institucional se relaciona con las capacidades organizacionales necesarias para alcanzar objetivos prefijados ^23^.

Para el análisis macro-institucional fueron considerados el contexto político, social y económico del país. Para analizar las capacidades micro-institucionales fueron utilizados para su operativización tres componentes del sistema de salud: modelo de atención, financiamiento e infraestructura ^24^.

El despliegue de las capacidades estatales se puede entorpecer debido a eventos y crisis externas, sin embargo, únicamente los resultados. Por lo tanto, sería posible observar un despliegue adecuado de capacidades mediante una efectiva gobernanza para obtener agilidad y resiliencia en el proceso ^11^, haciendo viable el análisis durante el contexto de la COVID-19.

Las técnicas del estudio involucraron el levantamiento de informaciones de: (1) página *web* del MSPAS de Guatemala; (2) informaciones complementarias obtenidas vía información pública; (3) reportes oficiales producidos por el Ministerio de Economía y Banco de Guatemala; (4) datos secundarios disponibles en bases de agencias internacionales como el Banco Mundial, Organización Mundial de la Salud y otras organizaciones como Our World in Data u observatorios nacionales de salud. El contexto social, económico y sanitario durante la pandemia fueron obtenidos de datos secundarios y complementados con información oficial.

El último estudio identificado realizó serie temporal del presupuesto del MSPAS de 1985 a 2008 ^25^. Esto permitió delimitar la colecta de datos del 2008 al 2021. Los valores fueron descriptos en quetzales (GTQ). El análisis de datos financieros consideró la deflación de los valores según el deflactor del producto interno bruto (PIB) del año 2021.

Para realizar el análisis de confiabilidad del presupuesto se utilizaron los tres últimos ejercicios fiscales de la colecta de informaciones: 2019 a 2021. En consonancia con la herramienta para la mejora de las prácticas de gestión de las finanzas públicas: Gasto Público y Rendición de Cuentas (PEFA, por sus siglas en inglés). Se evaluaron dos dimensiones: resultados del gasto agregado y resultados en la composición del gasto por clasificación funcional. Este mismo periodo permite realizar un análisis desagregado por dirección de área de salud y hospitales.

La descripción de brechas de infraestructura por área de salud corresponde al año de 2021, el más reciente, según datos oficiales del MSPAS. Debido a que la información utilizada proviene de fuentes públicas, no fue necesario el envío al comité de ética.

## Resultados

Para dar cuenta del objetivo del artículo se realiza una breve descripción inicial de la respuesta dada a la pandemia por COVID-19. Posteriormente, los resultados del estudio se organizan en dos secciones: el entorno macro-institucional y el micro-institucional.

### Contextualización de la respuesta a la pandemia por COVID-19 en Guatemala

En el Cuadro 1, se presentan algunos datos sobre la respuesta a la pandemia en Guatemala. Cabe destacar que los datos sobre el total de casos confirmados, el total de muertes y su letalidad están afectadas por las bajas tasas de cobertura de las pruebas realizadas (284,56 por mil habitantes). Así mismo, la vacunación dio inicio a finales de febrero de 2021, pero no alcanzó ni la mitad de la población meta vacunada con la primera dosis. Esto, acompañado de dificultades logísticas de distribución de vacuna, que llevó al almacenamiento de millones de dosis que perdieron validez antes de ser distribuidas. El sistema oficial no identificó el papel del subsistema comunitario.

**Cuadro 1:** Contexto social, económico, ambiental y político durante el contexto de COVID-19 en Guatemala, 2019 y 2022.

DIMENSIÓN	VARIABLES	2019	2022
Contexto social, económico y ambiental ^26^ ^,^ ^27^ ^,^ ^28^ ^,^ ^29^ ^,^ ^30^ ^,^ ^31^ ^,^ ^73^	Población total según el INE (número de habitantes)	16.604.026	17.357.886
	Índice de riesgo climático global (*ranking*)	65,67 * (62/180)	ND
	Índice de vulnerabilidad al cambio climático en América Latina y Caribe	0,75 (extremo)	ND
	IDH	0,663/127	0,629/136
	Pobreza total (%)	45,6	55,2
	Prevalencia de inseguridad alimentaria grave (%)	18,1	21,1
	Prevalencia de inseguridad alimentaria moderada a grave (%)	45,2	59,8
	Prevalencia del retraso del crecimiento en niños menores de 5 años (%)	44,9	43,5
	Déficit de vivienda	1,6 millones	2,2 millones
Contexto político ^30^ ^,^ ^31^	Régimen democrático híbrido		
	Índice de democracia ** (*ranking*)	5,26 (93/167)	4,62 (99/167)
	Proceso electoral y pluralismo **	6,92	6,92
	Funcionamiento del gobierno **	4,64	3,93
	Participación política **	3,89	3,89
	Cultura política **	4,38	2,50
	Libertades civiles **	6,47	5,59
	Índice de libertad de prensa	116/180	124/180
Contexto de COVID-19 (al 17 de junio de 2022)	Primer caso confirmado		14 de marzo de 2020
	Primera muerte confirmada		21 de marzo de 2020
	Número total de casos		878.401
	Casos por 100.000 habitantes		5.210,5
	Número total de muertes		18.371
	Muertes por millón de habitantes		1.006,64
	Tasa de letalidad		2,09%
	Total de pruebas realizadas		4.797.272
	Pruebas por 1.000 habitantes		284,56
	Fecha de inicio de vacunación		25 de febrero de 2021
	Personas vacunadas con primera dosis		8.283.105 (45,4%)
	Personas vacunadas con esquema completo		6.303.878 (34,5%)
	Dosis de refuerzo		3.042.031 (16,7%)
	Total de vacunas almacenadas ***		8.027.720 dosis
	Vacunas almacenadas con pérdida de fecha de validez ***		6.859.864 dosis
	Vacunas almacenadas disponibles para distribución ***		1.167.856 dosis

IDH: Índice de Desarrollo Humano; INE: Instituto Nacional de Estadística; ND: no disponible.

Fuente: elaboración própria, a partir de datos del Ministerio de Salud y Asistencia Social ^74^, Our World in Data ^75^ y fuentes citadas en el cuadro.

* Dato correspondiente a 2014;

** Escala de 0 a 10;

*** Datos al 17 de mayo de 2022.

### Entorno macro-institucional de Guatemala

Guatemala es una república, democrática y representativa. El territorio se divide en 22 departamentos y 340 municipios. Los municipios son autónomos en lo económico, técnico y administrativo. El sistema Legislativo es unicameral y conformado por 160 diputados electos. El Ejecutivo tiene un mandato improrrogable de cuatro años y las diputaciones, alcaldías, concejales y síndicos el mismo periodo, pudiendo ser reelectos.

En Guatemala existen condiciones políticas, sociales, económicas y ambientales ^26^ que colocan en vulnerabilidad a la población con altos números de pobreza ^27^ ^,^ ^28^, inseguridad alimentaria y bajo desarrollo humano ^29^, situación que se encrudece por la pandemia de COVID-19, cuando se comparan los datos disponibles en 2019 y 2022 (Cuadro 1). Inclusive Guatemala es uno de los países de América Latina con vulnerabilidad extrema al cambio climático con restringida capacidad de actuar, al mismo tiempo que existe un déficit de vivienda en constante aumento (2,5% anual).

El contexto político guatemalteco estudiado demuestra un deterioro democrático, con disminución en las dimensiones de funcionamiento del gobierno y de las libertades civiles ^30^, incluyendo las libertades de prensa ^31^ y un contexto de persecución política a jueces y defensores de la Madre Tierra. Por estas características, el régimen democrático guatemalteco se considera híbrido.

Durante la pandemia, el uso de la fuerza y la militarización en Guatemala fue una constante, mediante el uso de estados de excepción, inclusive en áreas con bajas tasas de transmisión por COVID-19, pero asociadas a la presencia de actividad minera o explotación de recursos florestales ^32^.

La concentración del poder económico y político configura un patrón de exclusión social y represión de la organización social producto de tres ciclos de reestructuración económica: el primero, el modelo híbrido agrícola-industrial (1950-1979), caracterizado por ser un periodo de crecimiento económico estable con políticas represivas militares y proletarización de la fuerza laboral, el segundo, la etapa de transición (1980-1986), periodo de crisis económica con impacto en el sector secundario y graves violaciones a los derechos humanos para la contención social. Por último, el modelo híbrido tripartito (1987-2019), en el cual se produce mayor concentración económico-política, la profundización de políticas neoliberales de privatización, desregulación y liberalización con aumento de pobreza y trabajo precario ^33^.

### Entorno micro-institucional: modelo de atención, financiamiento e infraestructura

#### Breve descripción del MSPAS

El MSPAS constituye el ministerio del Poder Ejecutivo encargado de la política sanitaria en el país. Constituye el subsistema público del sistema de salud. Está organizado en el despacho ministerial y cuatro viceministerios: Viceministerio de Hospitales, Atención Primaria en Salud, Regulación, Vigilancia y Control de la Salud y el Administrativo Financiero.

Las estructuras gerenciales están conformadas por el nivel central del MSPAS. De este, la Dirección General del Sistema de Integral de Atención en Salud posee a su cargo las Direcciones de Área de salud (DAS) que tienen a su cargo los Distritos Municipales de Salud.

El sistema se organiza en niveles de atención. El primer nivel de atención lo conforman las unidades más cercanas a la población siendo los centros comunitarios y puestos de salud atendidos por auxiliares de enfermería y los centros de salud, dónde hay personal médico y de enfermería. El segundo nivel de atención está conformado por el Centro de Atención Permanente (CAP), Centro de Atención Materno Infantil (CAIMI), Hospitales Generales Tipo I y Centros de Atención a Especialidades. El tercer nivel está conformado por hospitales tipo II, III y IV.

#### Modelo de atención

El modelo de atención en salud en Guatemala está condicionado por múltiples reformas. En el periodo autoritario del estado desarrollista (1954-1985), estas fueron de carácter aditivo y parcial. En la transición democrática (1985-1990) se retoma la salud como deber de Estado, aunque la influencia de organismos internacionales instaura programas verticales en línea con una APS selectiva. Posteriormente, en el periodo de reforma sectorial (1991-1999) se impulsa el Programa de Extensión de Cobertura (PEC). Este consistía en oferta de paquetes básicos de servicios de salud a cargo de organizaciones no gubernamentales (ONG) contratadas por el ministerio con servicios itinerantes, por su parte, la atención permanente estaba a cargo de facilitadores comunitarios y vigilantes de la salud. La rectoría, vigilancia y regulación en salud no se fortalecieron ^25^.

En el periodo de 2000-2009 se elimina el copago, seguido de una revigorización del PEC y la recategorización nominal de servicios de salud de segundo nivel. En 2013 se prohibió la contratación de ONG en el primer nivel de atención, los recursos son destinados al MSPAS en un contexto de desfinanciamiento, desabastecimiento y políticas clientelistas de contratación ^34^.

En el año 2016, se inicia un ejercicio de territorialización y sectorización que tendría como objetivo crear condiciones para un modelo de atención basada en la APS ^35^, esto da paso a la conformación de un viceministerio específico. Este modelo fue derogado, en su lugar se promulgaron las redes integradas de servicios de salud y un modelo de atención y gestión. En 2020, fueron implementados seguros escolares para la atención de las infancias matriculadas en escuelas.

A pesar de las estrategias implementadas, los niveles de atención no poseen mecanismos de continuidad definidos. Tanto en el primero como en el segundo nivel de atención, ésta ocurre por demanda espontánea y los centros de atención a especialidades no han sido implementados. Persiste la segmentación y fragmentación del sistema con escasa participación del subsector comunitario de atención en salud de los pueblos que conforman Guatemala: Garífuna, Xinca, Maya y Mestizo. El género es mencionado en documentos institucionales sin su clara operativización.

#### Financiamiento

El gasto como porcentaje del PIB ha sido en promedio 1,29% desde el 2008, con un aumento progresivo de 1,40% a 1,99% entre los años de 2019 y 2021. El aumento del gasto por la crisis sanitaria de COVID-19 se dio mediante modificaciones presupuestarias (21,5% en 2020 y 34,5% en 2021) con baja ejecución (86,51%) (Tabla 1).

**Tabla 1:** Presupuesto asignado, modificaciones presupuestarias y presupuesto vigente del Ministerio de Salud de Guatemala (en quetzales -GTQ) y como porcentaje del producto interno bruto (PIB), 2008-2021.

Año	Asignado corrientes (GTQ)	Modificaciones corrientes (GTQ)	Vigente corrientes (GTQ)	Ejecución (%)	Asignado constantes (GTQ)	Vigente constantes (GTQ)	Gasto/PIB (%)
2008	3.000.031.879,00	-235.650.000,00	2.764.381.879,00	97,11	4.593.355.602,74	4.232.551.354,17	0,93
2009	3.737.700.344,00	-395.823.895,00	3.341.876.449,00	96,82	5.524.183.755,69	4.939.170.584,59	1,09
2010	3.737.700.344,00	102.390.712,20	3.840.091.056,20	93,40	5.254.126.571,51	5.398.058.324,23	1,15
2011	3.929.634.505,00	524.097.293,00	4.453.731.798,00	89,29	5.167.433.684,48	5.856.616.864,83	1,20
2012	4.434.953.997,00	-22.606.947,00	4.412.347.050,00	95,81	5.641.444.841,46	5.612.687.870,22	1,12
2013	5.111.600.000,00	96.090.664,00	5.207.690.664,00	94,77	6.290.846.120,00	6.409.104.900,18	1,23
2014	5.111.600.000,00	662.668.909,00	5.774.268.909,00	87,73	6.115.930.507,49	6.908.801.036,66	1,27
2015	5.647.224.460,00	915.000.000,00	6.562.224.460,00	83,99	6.609.014.019,52	7.679.849.413,20	1,34
2016	5.531.691.485,00	857.099.221,00	6.388.790.706,00	92,83	6.303.567.324,62	7.280.263.631,37	1,22
2017	6.897.096.196,00	0	6.897.096.196,00	86,13	7.724.320.946,78	7.724.320.946,78	1,24
2018	6.897.096.196,00	108.243.293,00	7.005.339.489,00	91,77	7.633.324.000,38	7.753.121.680,86	1,19
2019	8.197.157.000,00	80.400.000,00	8.277.557.000,00	95,39	8.750.317.564,32	8.836.143.117,27	1,40
2020	8.197.157.000,00	1.766.000.000,00	9.963.157.000,00	86,51	8.524.793.915,75	10.361.380.192,58	1,66
2021	9.823.157.000,00	3.422.226.190,00	13.245.383.190,00	85,84	9.823.157.000,00	13.245.383.190,00	1,99

Fuente: elaboración própria, a partir de datos del Ministerio de Salud y Asistencia Social ^74^ y Banco de Guatemala ^76^.

Cuando se observa el gasto destinado a la APS (Tabla 2) se observa que representa aproximadamente un cuarto del presupuesto total. En 2021 disminuye 3%. Al desagregar por áreas de salud Santa Rosa, Jutiapa y Baja Verapaz poseen el mayor gasto per cápita. Áreas de salud con altos niveles de pobreza multidimensional y bajo Índice de Desarrollo Humano poseen valores por debajo de la media de gasto destinado a primer y segundo nivel de atención, por ejemplo, Alta Verapaz y Quiché.

**Tabla 2:** Gasto per cápita en atención primaria en salud (APS) por Dirección de Área de Salud e Índice de Desarrollo Humano, porcentaje (%) del gasto en APS sobre el gasto total y como gasto destinado a la APS del 2019-2021, Guatemala.

Dirección de Área de Salud	IDH	2019			2020			2021		
		Gasto per cápita *	% del gasto total **	% de la APS ***	Gasto per cápita *	% del gasto total **	% de la APS ***	Gasto per cápita *	% del gasto total **	% de la APS ***
Guatemala Nororiente	0,614	192,1	1,1	4,0	226,6	1,1	4,2	269,3	1,1	4,2
Guatemala Noroccidente	0,614	116,1	1,1	3,9	127,4	1,0	3,8	156,9	1,0	4,0
Guatemala Sur	0,614	48,9	0,6	2,0	58,0	0,6	2,1	67,2	0,5	2,1
El Progreso	0,518	185,3	0,4	1,3	195,6	0,3	1,2	257,8	0,3	1,4
Sacatepéquez	0,567	116,0	0,5	1,6	138,2	0,5	1,7	188,8	0,5	2,0
Chimaltenango	0,487	128,6	1,0	3,6	133,8	0,9	3,3	152,6	0,8	3,3
Escuintla	0,516	116,9	1,0	3,6	138,6	1,0	3,8	163,6	1,0	3,8
Santa Rosa	0,47	298,2	1,4	4,8	345,4	1,3	4,9	366,6	1,1	4,5
Sololá	0,455	218,8	1,2	4,3	229,6	1,1	4,0	281,1	1,0	4,2
Totonicapán	0,432	180,7	1,1	3,9	196,4	1,0	3,7	225,5	0,9	3,7
Quetzaltenango	0,529	121,7	1,2	4,4	133,5	1,2	4,2	161,5	1,1	4,4
Suchitepéquez	0,471	102,1	0,7	2,3	121,4	0,7	2,5	133,0	0,6	2,3
Retalhuleu	0,476	133,3	0,5	1,8	157,9	0,5	1,9	208,7	0,5	2,1
San Marcos	0,451	170,3	2,2	7,6	197,4	2,1	7,8	225,1	1,9	7,6
Huehuetenango	0,399	157,6	2,4	8,3	172,6	2,2	8,0	188,9	1,9	7,6
Quiché	0,424	210,8	1,7	6,0	238,0	1,6	6,0	271,7	1,5	5,9
Ixcán	0,424	538,8	0,6	2,1	602,5	0,6	2,0	699,7	0,5	2,0
Baja Verapaz	0,457	236,2	0,8	2,8	263,2	0,8	2,8	296,6	0,7	2,7
Alta Verapaz	0,37	149,6	2,2	7,6	163,0	2,0	7,3	183,9	1,8	7,1
Petén Norte	0.458	174,9	0,3	1,2	200,3	0,3	1,2	248,4	0,3	1,3
Izabal	0,481	138,5	0,7	2,6	164,8	0,7	2,7	191,3	0,7	2,7
Zacapa	0,511	178,1	0,5	1,8	200,7	0,5	1,8	276,5	0,5	2,1
Chiquimula	0,408	212,0	1,0	3,5	230,9	0,9	3,4	254,3	0,8	3,2
Jalapa	0,426	173,5	0,7	2,4	203,7	0,7	2,5	219,7	0,6	2,3
Jutiapa	0.455	276,7	1,5	5,2	328,3	1,5	5,5	377,9	1,4	5,5
Petén Suroccidente	0,458	235,5	0,5	1,8	272,1	0,5	1,9	301,0	0,4	1,8
Petén Suroriente	0,458	306,0	0,6	2,0	363,1	0,6	2,1	391,9	0,5	1,9
Guatemala Central	0,614	54,7	0,6	2,1	67,8	0,7	2,4	93,4	0,7	2,8
Ixil	0,424	279,22	0,48	1,7	299,2	0,4	1,6	356,1	0,4	1,6
Gasto APS nacional		156,24	28,41	ND	176,6	27,4	ND	205,3	24,9	ND

IDH: Índice de desarrollo humano; ND: no disponible.

Fuente: elaboración própria, a partir de datos del Ministerio de Salud y Asistencia Social (MSPAS) ^74^ y Banco de Guatemala ^76^.

* Cálculo realizado con base a población registrada por cada área de salud del MSPAS en 2017;

** Gasto en APS/Gasto total del MSPAS (%);

*** Gasto en APS/Gasto total en APS del MSPAS (%).

El presupuesto asignado a hospitales ocupa la mitad del gasto total del MSPAS, dirigido especialmente a los hospitales de referencia nacional (32,24%). Mientras tanto, el total destinado a hospitales regionales y departamentales es inferior, así como su ejecución presupuestaria (Tabla 3).

**Tabla 3:** Infraestructura hospitalaria por tipo de hospital y presupuesto como porcentaje del gasto total del Ministerio de Salud y Asistencia Social (MSPAS) y como porcentaje del gasto asignado a hospitales en Guatemala y porcentaje de ejecución 2019 al 2021.

Hospital	2019			2020			2021		
	% del gasto total *	% del hospital **	% de ejecución ***	% del gasto total *	% del hospital **	% de ejecución ***	% del gasto total *	% del hospital **	% de ejecución ***
Hospitales especializados									
Hogar de Ancianos Fray Rodrigo De La Cruz	0,2	0,4	98,7	0,2	0,3	94,9	0,1	0,3	94,3
Salud Mental Dr. Federico Mora	0,9	1,8	97,2	0,8	1,6	96,0	0,7	1,4	96,8
Ortopedia y Rehabilitación Dr. Jorge Von Ahn	0,4	0,7	97,2	0,4	0,7	91,9	0,3	0,6	91,5
Infantil de Infectología y Rehabilitación	0,6	1,2	95,8	0,6	1,2	89,3	0,5	1,1	88,1
San Vicente	0,5	1,0	95,4	0,5	1,0	93,3	0,5	1,1	84,6
Especialidades Rodolfo Robles	0,3	0,7	95,7	0,3	0,6	89,5	0,3	0,7	78,5
Infantil Elisa Martínez, Puerto Barrios, Izabal	0,5	0,9	95,6	0,5	1,0	89,6	0,4	0,9	86,5
Villa Nueva	1,1	2,3	84,3	2,0	4,0	60,4	2,2	4,8	71,3
Hospitales nacionales									
General San Juan de Dios	9,3	18,6	99,2	8,1	16,3	89,1	7,3	15,7	90,5
Roosevelt	9,6	19,2	95,7	8,5	17,1	89,2	8,0	17,2	95,8
Hospitales regionales									
Regional de Cuilapa Licenciado Guillermo Fernández Ller	1,9	3,7	97,7	1,7	3,4	90,8	1,8	3,8	85,5
Regional de Occidente Quetzaltenango	2,9	5,9	98,3	3,5	7,1	84,5	3,2	6,8	95,1
Regional de Huehuetenango Dr. Jorge Vides Molina	1,4	2,9	94,8	1,5	3,1	77,5	1,5	3,2	87,1
Regional de El Quiché	1,0	1,9	89,7	0,9	1,8	78,4	1,0	2,1	90,6
Regional de Cobán	1,1	2,2	88,2	1,1	2,1	81,6	1,0	2,2	82,6
Regional de Zacapa	1,4	2,8	86,3	1,6	3,3	80,0	1,4	3,0	80,3
Hospitales departamentales									
El Progreso	0,4	0,7	96,3	0,4	0,7	92,0	0,4	0,8	83,4
Pedro de Bethancourt	1,5	3,0	99,1	1,5	3,1	90,0	1,5	3,2	90,6
Escuintla	1,7	3,5	95,3	1,9	3,8	88,2	1,6	3,4	88,8
San Marcos Dr. Moisés Villagrán Mazariegos	0,9	1,8	98,8	0,8	1,7	94,6	0,8	1,7	92,2
Sololá	0,6	1,2	89,9	0,6	1,1	89,9	0,6	1,3	77,4
Totonicapán	0,8	1,6	86,2	0,8	1,6	83,2	0,8	1,8	87,0
Salamá	0,5	1,0	86,4	0,5	1,1	78,2	0,4	0,9	89,8
Nicolasa Cruz Jalapa	0,6	1,1	97,5	0,6	1,1	93,5	0,6	1,3	87,8
Chiquimula	0,7	1,5	96,1	0,8	1,6	89,7	0,8	1,6	91,7
Chimaltenango	1,0	2,0	98,4	0,9	1,8	97,2	0,8	1,7	88,3
Mazatenango	0,9	1,7	96,7	0,9	1,7	89,9	0,8	1,6	92,2
San Benito	1,0	2,0	97,8	1,5	2,9	87,8	1,3	2,7	90,5
Retalhuleu	0,6	1,2	97,2	0,7	1,3	91,4	0,7	1,4	90,7
Amistad Japón Guatemala	0,9	1,9	97,9	1,0	2,0	85,6	0,8	1,7	96,6
Ernestina Garcia Vda. de Recinos	0,9	1,9	96,4	0,9	1,8	85,3	0,8	1,7	91,3
Hospitales distritales									
Tipo I de Tecpán Guatemala	0,0	0,0	0,0	0,2	0,4	87,0	0,2	0,4	91,3
Barillas	0,2	0,5	87,5	0,3	0,5	90,9	0,2	0,5	89,5
Tiquisate	0,4	0,9	97,5	0,4	0,8	91,6	0,3	0,7	93,4
Sayaxché Petén	0,3	0,6	98,9	0,3	0,6	94,5	0,3	0,6	97,2
Malacatán San Marcos	0,5	1,0	94,4	0,5	1,0	84,9	0,5	1,0	78,8
La Tinta	0,3	0,6	55,7	0,2	0,4	62,9	0,2	0,4	77,4
Coatepeque	1,1	2,1	98,7	0,9	1,7	96,0	0,8	1,8	92,9
Fray Bartolomé De Las Casas	0,2	0,5	94,1	0,3	0,5	82,4	0,2	0,5	95,2
Amatitlán	0,7	1,5	97,1	0,8	1,6	91,0	0,7	1,5	94,0
San Pedro Necta	0,2	0,4	93,2	0,2	0,4	93,8	0,2	0,3	92,6
Poptún	0,4	0,8	96,6	0,4	0,7	92,9	0,4	0,8	86,2
Melchor de Mencos	0,3	0,6	95,6	0,3	0,5	90,2	0,2	0,5	88,5
Uspantán	0,1	0,3	96,2	0,1	0,3	90,0	0,1	0,3	82,2
Nebaj	0,2	0,4	97,5	0,3	0,5	58,4	0,3	0,5	75,2
Joyabaj	0,1	0,3	90,9	0,3	0,6	42,7	0,1	0,3	75,4
Total de hospitales	51,0	100,0	95,7	51,1	100,0	86,6	47,1	100,0	89,5

Fuente: elaboración própria, a partir de datos del Ministerio de Salud y Asistencia Social (MSPAS) ^74^ ^,^ ^77^ y Banco de Guatemala ^76^.

* Gasto del hospital/Gasto total del MSPAS (%);

** Gasto del hospital/Gasto total en hospitales del MSPAS (%);

*** Porcentaje de ejecución del presupuesto.

El grado de confiabilidad del presupuesto de los tres últimos periodos fiscales refleja una variabilidad del 95% a 85% lo cual le coloca en la clasificación C de confiabilidad, con una varianza de 10%. Las reasignaciones presupuestarias modificaron la composición del gasto durante el ejercicio fiscal, mostrando un grado B para el indicador.

#### Infraestructura

Según datos del MSPAS ^36^ al 2021 el 53,98% de sectores no poseen infraestructura (Tabla 4). El área metropolitana posee brechas que superan el 90%. Diecisiete áreas de salud poseen brechas arriba del 50%.

**Tabla 4:** Brechas de infraestructura de atención primaria en salud (APS) y establecimientos de segundo nivel de atención y brechas de sectores sin infraestructura al 2021, Guatemala.

Dirección de Área de Salud	Territorialización		Sectores sin infraestructura				Establecimientos de APS			Establecimientos del segundo nivel de atención		
	Territorio (n)	Sector (n)	Rural (n)	Urbano (n)	Total (n)	%	CC (n)	PS (n)	CS (n)	CAP (n)	CAIMI (n)	CENAPA (n)
Guatemala Central	96	333	42	269	311	93,39	0	6	13	1	0	0
Guatemala Sur	114	380	40	305	345	90,79	15	15	5	3	0	0
Guatemala Noroccidente	100	314	84	174	258	82,17	8	28	7	4	0	0
Guatemala Nororiente	54	160	55	70	125	78,13	1	30	4	4	0	0
Escuintla	76	252	76	111	187	74,21	8	38	8	7	0	0
Huehuetenango	158	617	344	110	454	73,58	420	111	7	19	2	5
Suchitepéquez	60	243	83	91	174	71,60	59	27	7	5	0	3
Sacatepéquez	40	139	27	68	95	68,35	59	27	7	5	0	3
Peten Norte	23	88	24	36	60	68,18	9	16	4	0	0	0
Totonicapán	65	237	115	33	148	62,45	64	28	1	6	1	1
Retalhuleu	37	162	48	52	100	61,73	38	38	10	1	0	0
Ixcán	12	47	26	3	29	61,70	29	13	0	3	1	0
Peten Sur Oriente	28	76	32	10	42	55,26	1	28	6	0	0	0
Jutiapa	58	221	77	44	121	54,75	54	54	2	9	1	7
Quetzaltenango	98	401	115	101	216	53,87	150	70	12	9	1	5
Izabal	50	177	58	37	95	53,67	3	32	4	1	1	0
Chimaltenango	72	262	60	71	131	50,00	101	49	14	3	0	0
Sololá	62	170	46	32	78	45,88	61	39	0	19	0	0
Quiche	94	346	115	40	155	44,80	140	57	4	15	0	0
San Marcos	146	600	176	74	250	41,67	481	80	11	16	2	0
Zacapa	27	110	22	23	45	40,91	6	53	10	1	0	0
Santa Rosa	52	208	47	28	75	36,06	47	53	11	2	1	0
Jalapa	39	141	31	15	46	32,62	51	32	4	4	0	0
El Progreso	20	76	9	15	24	31,58	0	39	6	2	0	0
Chiquimula	53	227	25	37	62	27,31	90	29	7	4	0	0
Ixil	23	108	2	19	21	19,44	65	32	0	3	0	0
Baja Verapaz	35	164	8	17	25	15,24	34	117	3	5	0	0
Alta Verapaz	167	670	38	60	98	14,63	481	39	2	14	1	1
Peten Sur Occidente	16	66	1	5	6	9,09	7	67	5	1	0	0
Total	1.875	6.995	1.826	1.950	3.776	53,98	2.482	1.247	174	166	11	25

CAIMI: Centro de Atención Materno Infantil; CAP: Centro de atención permanente; CENAPA: Centro de Atención a Pacientes Ambulatorios; CC: Centro Comunitario; CS: Centro de Salud; PS: Puesto de Salud.

Fuente: elaboración própria, a partir de datos del Ministerio de Salud y Asistencia Social ^74^.

Por tipo de establecimiento, predominan los Centros Comunitarios de Salud, contrario a los puestos de salud de carácter formal. Los centros de salud no poseen brecha estimada debido a la ausencia de un estimado de adscripción poblacional.

La red hospitalaria está compuesta por 46 hospitales (Tabla 3), de los cuales dos son de referencia nacional y ocho hospitales especializados. Durante la pandemia fueron habilitados cinco hospitales temporales: Parque de la Industria, Quetzaltenango, Escuintla, Petén y Zacapa.

## Discusión

### Entorno macro-institucional de Guatemala

El contexto social en Guatemala es un importante limitador de la capacidad institucional para sostener una política social y sanitaria democrática. Los procesos de reproducción social de salud-enfermedad vigentes limitan la construcción de políticas e instituciones de salud ^37^ que persigan como objetivo una vida saludable. Países con mejores condiciones de vida fueron más eficaces en abordar la pandemia, contrario a las disparidades observadas en poblaciones históricamente marginalizadas ^38^.

En el contexto guatemalteco la capacidad estatal tiende a perseguirse mediante el uso de la fuerza, sin embargo, esto no genera legitimidad ^39^ al igual que la militarización en otros países latinoamericanos ^40^. Por el contrario, un mayor grado de legitimidad y aceptación de las decisiones crea un ambiente positivo para el actuar público ^41^.

Dado el carácter autocrático en Guatemala ^42^, la participación social fue limitada. Consecuentemente, no existen condiciones propicias para el fortalecimiento institucional ante su poca legitimidad. Países con ambientes democráticos consolidados tuvieron mejores resultados durante la COVID-19, con implementación de políticas de valorización de la vida ^43^, defensa de la protección de las libertades y amplia participación social ^8^.

El desempeño de las democracias gana legitimidad mediante la distribución de bienes públicos, sin embargo, en contextos democráticos débiles se observa un aparato burocrático con regímenes patrimoniales y corrupción ^43^. Para Grindle ^44^, un proceso de estagnación o declive genera un proceso de crisis entre el Estado y la sociedad civil. De hecho, en Guatemala, los procesos de exclusión pasan por mecanismos económicos de dominación que limitan el despliegue de capacidades institucionales.

La creciente concentración del poder económico-político de la élite empresarial, de grupos nacionales y corporaciones multinacionales ha generado un Estado mínimo incapaz de producir una política para la felicidad de sus habitantes. Argumentos variados intentan reforzar el Estado mínimo y políticas sociales regresivas ^42^ ignorando su efecto multiplicador ^45^. Durante la pandemia, Estados con sistemas de protección social débiles fueron los que gastaron más para compensar sus fallas, por el contrario, los países con sistemas fuertes se ocuparon en fortalecer y extender las políticas sociales ^46^ ^,^ ^47^.

La historia de la política sanitaria (Cuadro 1), desnuda el no compromiso con la construcción de un proyecto sanitario, ni desde la perspectiva biomédica para garantizar la restauración funcional, tampoco, de un proyecto pautado en la salud colectiva. Además, de las políticas aditivas, reactivas y sin participación popular que han caracterizado las políticas en Guatemala, se observan reformas de carácter neoliberales, coincidentes con el tercer ciclo de reestructuración económica, con efectos severos de creación de condiciones para garantizar el derecho a la salud, con invisibilidad o un uso instrumental del componente cultural y de género.

### Entorno micro-institucional: modelo de atención, financiamiento e infraestructura

#### Modelo de atención

El modelo de atención constituye un factor que limita la atención integral de la población guatemalteca, toda vez existe poca delimitación debido a reformas aditivas y parciales. Se entiende aquí al modelo de atención como la lógica o racionalidad que orienta la combinación tecnológica en las prácticas de salud, es decir, las intervenciones de salud con base a las necesidades individuales, familiares o comunitarias ^24^.

La racionalidad histórica dada por las múltiples modificaciones y discontinuidades provoca brechas de capacidades del MSPAS en su organización interna, tanto en la distribución de funciones y delimitaciones de flujos de información y decisión, como en la existencia de sistemas de infraestructura y financiamiento. Así como la generación de capacidades individuales.

En Guatemala, no se ha priorizado la APS. Existe sobrecarga a trabajadoras y trabajadores de salud, principalmente auxiliares de enfermería, con demandas de atención basadas en programas verticales y funciones que extrapolan la formación de corta duración que poseen ^48^. El proceso de trabajo responde a la demanda espontánea con foco en el daño. Según Paim ^49^, un modelo de esta naturaleza refuerza las inequidades, ya que, estimula las fuerzas expansionistas del mercado y limita la posibilidad de una atención con equidad. La contracción de los sistemas de salud públicos y la ausencia de enfoques basados en APS limitaron la capacidad de asistencia durante la pandemia ^6^ ^,^ ^50^, al igual la segmentación y la fragmentación de los sistemas de salud ^51^.

La resolutividad del sistema se ve afectado debido a la ausencia de equipos multidisciplinares o de apoyo matricial. La baja capacidad resolutiva se visualiza igualmente en el segundo nivel de atención. Los hospitales departamentales, ante la ausencia de materiales y equipamientos adecuados, derivan pacientes hacia los hospitales localizados en la región metropolitana. Consecuentemente, la red hospitalaria de referencia nacional no logra cumplir sus funciones de cuidados especializados de alta densidad tecnológica, debido a la sobrecarga recibida ante la falta de una organización en red y el constante desabastecimiento de insumos.

El enfoque del modelo de atención se concentra en padecimientos agudos y problemas materno-infantiles, lo cual limita el acceso a personas que conviven con condiciones crónicas ^52^. Esta población se enfrenta a la falta de continuidad y longitudinalidad del cuidado al no considerarse puntos de atención específicos para cuidados de proximidad. Los cuidados intermediarios poseen el potencial de aumentar el grado de acciones posibles de la APS y garantizar mayor resolutividad del sistema ^53^, sin embargo, son poco discutidos.

A pesar de Guatemala ser un país conformado por cuatro pueblos: Garífuna, Xinca, Maya y Mestizo, la relación entre el modelo occidental de atención y los modelos de atención indígenas continúa siendo un desafío. Dificulta el racismo estructural que influye en la conformación de la red de servicios ^35^, la ausencia de políticas de formación y carrera en la función pública para talento humano de las comunidades, que hablen el idioma local e impulsar la relación entre diferentes saberes. El reconocimiento de la validez epistémica ancestral es fundamental para el acceso a servicios de salud resolutivos e integrales ^54^.

Durante la pandemia se hizo evidente el impacto de las diferentes expresiones de espiritualidad en el acceso a servicios de salud y a tecnologías específicas de prevención ^55^, que ya venía siendo discutido en trabajos previos ^54^, sin embargo, el modelo no contempla las mejores estrategias para generar competencia cultural y religiosa para evitar barreras simbólicas a los servicios de salud, al cual la accesibilidad ya es reducida. Igualmente, con la dificultad de transversalizar la perspectiva de género en sus estrategias.

#### Financiamiento

Como señalado en la sección, el modelo de atención influencia el modelo organizacional y gerencial, y la articulación existente entre los diferentes establecimientos de salud ^24^, de tal forma, se analizan las limitaciones de las capacidades institucionales en el componente financiamiento.

En Guatemala, históricamente, las capacidades del MSPAS se han visto mermadas por su desfinanciamiento histórico. Es fundamental contar con políticas sociales suficientemente financiadas para dotar de capacidades a las instituciones. Aunque, este no explique por completo la eficacia de su implementación ^56^.

El gasto como porcentaje del PIB tuvo un leve aumento. Una serie anterior, de 1985 a 2008, muestra que la inversión en salud apenas llegó a 1% del PIB ^25^. El aumento del gasto durante la crisis sanitaria de COVID-19 se dio mediante modificaciones presupuestarias, sin embargo, la capacidad de ejecución fue baja. Esto muestra que un aumento financiero no necesariamente se traduce en mayor capacidad, debido a que otros factores juegan un papel fundamental tales como la cultura organizacional, coordinación, tecnologías y logística ^56^. La pérdida de vacuna demostró la incapacidad de generar modelos de distribución logísticos, aunque representó una inversión significativa.

El análisis de tres periodos fiscales (2019-2021) permite evaluar el grado de confiabilidad del presupuesto. En casos de crisis es posible la existencia de años atípicos, tal cual ocurrió en el 2020. Sin embargo, era esperado una recomposición del gasto en el siguiente año fiscal. El grado de confiabilidad del presupuesto indica que existe una práctica a ser mejorada en términos de planificación del gasto público en salud ^57^.

En otros contextos, la falta de confiabilidad ha evidenciado un amplio margen de maniobra sin mecanismos de pesos y contrapesos que garanticen la certidumbre sobre el gasto público ^57^. Procesos participativos para el seguimiento y evaluación de los fondos públicos tienden a tener mejores resultados ^58^.

Sistemas de salud, como instituciones sociales, poseen el potencial de combatir las inequidades sociales o reforzarlas ^59^. En el caso guatemalteco, parece reforzarlas debido al bajo financiamiento y la poca confiabilidad sobre el uso de los recursos públicos, reverberando en un alto costo para las familias para acceder a servicios de salud, en un contexto cruel por altos índices de pobreza, provocando barreras de acceso y gastos catastróficos en salud.

El gasto privado de bolsillo no solo proviene de la búsqueda directa de servicios privados, sino de pagos informales o indirectos al acudir a servicios públicos de salud. Por ejemplo, medicamentos, exámenes complementarios y combustible para ambulancias ^60^ ^,^ ^61^. De tal forma, la capacidad institucional financiera es limitada y aumenta las inequidades en un contexto macro-institucional adverso.

#### Infraestructura

La infraestructura sanitaria fortalece la capacidad institucional ^56^ y favorece el acceso a servicios de salud ^62^. Asimismo, poseer una infraestructura adecuada permite un ambiente propicio para desarrollar el proceso de trabajo de los equipos y aumentar su resolutividad ^63^ y la satisfacción de las personas usuarias ^64^.

Actualmente, Guatemala posee una capacidad instalada de infraestructura con distribución inequitativa que coloca barreras geográficas a la continuidad. Con limitada inversión se reduce la capacidad estatal de garantizar derechos ^62^, toda vez la composición público-privada se complejiza al promover el libre mercado, dificultando la expansibilidad de la APS ^65^.

El ejercicio de territorialización evidencia una brecha de infraestructura y talento humano para garantizar el acceso a servicios de APS ^36^, lo cual puede explicar la sobrecarga hospitalaria. La falta de infraestructura para una APS resolutiva y de base territorial, hace que los hospitales se ocupen de atender causas plausibles de ser resueltas en otros puntos de atención y dificulta su proceso de trabajo al sobrecargar el sistema. Además, los centros urbanos del área metropolitana poseen un abandono del enfoque de prevención y promoción de la salud ^66^.

Predominan los Centros Comunitarios de Salud, contrario a los puestos de salud de carácter formal con modelo de atención definido, reduciendo la APS a una cartera de servicios. Los establecimientos de segundo nivel poseen una brecha de aproximadamente 2.000 centros de salud ^67^. Esto impide llevar a cabo acciones de organización en red con mecanismos de comunicación definidos, generando itinerarios terapéuticos discontinuos ^68^.

La infraestructura hospitalaria posee deficiencias en términos de la cualidad de las obras. Actualmente, la red hospitalaria está compuesta por 46 hospitales con imposibilidad de ofrecer atención con mayor densidad tecnológica. A pesar de las barreras de accesibilidad geográfica, física, talento humano e infraestructura, estudios colocan como responsabilidad individual el abandono a tratamientos ^69^, contrario a modificaciones de corto, mediano y largo plazo para la mejoría de la capacidad institucional ^70^.

Durante la pandemia fueron abiertos cinco hospitales temporales. Estos tuvieron problemas para su funcionamiento con ausencia de padrones de calificación internacional y sustanciales demoras para mejorar su capacidad resolutiva ^71^. Esto evidencia la imposibilidad de generar infraestructura adecuada para el cuidado continuado y responder a eventos catastróficos con atención especializada de alto padrón. Además, imposibilita generar un espacio seguro para las personas trabajadoras y las personas usuarias con el equipo y tecnologías adecuadas en la infraestructura digna ^72^. La insuficiente capilaridad es un impedimento para el derecho a la salud.

## Consideraciones finales

En Guatemala existe una deficiente capacidad estatal para garantizar el derecho a la salud a su población y responder a crisis. La incapacidad de poseer un sistema resiliente se debe a la falta de fortalecimiento de las capacidades micro-institucionales en el contexto macro de la histórica privatización por desgaste. Por ejemplo, el modelo de atención y su racionalidad médico-hegemónica limita el actuar del sistema de salud, prioriza la actuación reactiva, principalmente, hospitalaria. El desfinanciamiento ha sido histórico y existe una infraestructura deficiente para atender la totalidad de la población. Debe ser fortalecida la capacidad de adaptarse y aprender de forma rápida, alineando los servicios públicos con las necesidades de la ciudadanía. Además, requiere el fortalecimiento de sus capacidades mediante un redireccionamiento claro de sus objetivos mediante un plan estratégico de desarrollo institucional.

Entender las características macro-institucionales es importante, ya que delinean las raíces de una estructura burocrática no perfeccionada que solo tiende a perpetuar la imposibilidad de actuar, reproduce inequidades y limita las posibilidades de transformación del sistema de salud en Guatemala. El contexto macro-institucional apunta la necesaria radicalización democrática para la recuperación del Estado y el combate al racismo estructural con equidad de género.

El escenario macro coloca los desafíos políticos, democráticos y de transparencia como fundamentales, sin embargo, se argumentó en el presente artículo que, además, existe una baja capacidad estatal a nivel micro-institucional dada la falta de direccionamiento claro sobre sus objetivos y resultados. A partir de la reflexión suscitada, se quiere realizar un llamado a realizar estudios empíricos sobre los diferentes componentes de las capacidades estatales y el fortalecimiento de la investigación en políticas, planificación y gestión sanitaria, así como una cultura de evaluación de políticas y programas para el MSPAS, con el objetivo de generar evidencias que sustenten el fortalecimiento de las capacidades institucionales.
